# Non-rhinovirus enteroviruses associated with respiratory infections in Peru (2005-2010)

**DOI:** 10.1186/1743-422X-11-169

**Published:** 2014-09-22

**Authors:** Jose L Huaman, Gladys Carrion, Julia S Ampuero, Jorge Gomez, Victor Ocaña, Irmia Paz, Elizabeth Gomez, Edward Chavez, Favio Sarmiento, Edward Pozo, V Alberto Laguna-Torres, Eric S Halsey

**Affiliations:** US Naval Medical Research Unit No. 6, Lima, Peru; Dirección General de Epidemiología, Ministerio de Salud, Lima, Peru; Centro de Salud Pachitea, Dirección Regional de Salud Piura, Ministerio de Salud, Piura, Peru; Facultad de Medicina, Universidad Nacional de San Agustín, Arequipa, Peru; Hospital Regional Manuel Nuñez Butrón, Dirección Regional de Salud Puno, Ministerio de Salud, Puno, Peru; Centro de Salud Militar, 32a Brigada de Infantería, Trujillo, Peru; Hospital Regional de Pucallpa, Dirección Regional de Salud Ucayali, Ministerio de Salud, Pucallpa, Peru; Dirección de Epidemiologia, Sub Región de Salud “Luciano Castillo Colona”, Ministerio de Salud, Sullana, Peru

**Keywords:** Enterovirus, Respiratory infections, Peru

## Abstract

**Background:**

Enteroviruses (EVs) are a common cause of respiratory tract infections and are classified into seven species (EVA-D and rhinoviruses [RHVs] A-C) with more than 200 different serotypes. Little is known about the role of non-RHV EVs in respiratory infections in South America. The aim of this study was to describe the epidemiology of non-RHV EVs detected in patients with influenza-like illness enrolled in a passive surveillance network in Peru.

**Methods:**

Throat swabs and epidemiological data were collected from participants after obtaining verbal consent. Viral isolation was performed in cell culture and identified by immunofluorescence assay. Serotype identification of EV isolates was performed using commercial monoclonal antibodies. Identification of non-serotypeable isolations was carried out by reverse transcriptase-PCR, followed by sequencing.

**Results:**

Between 2005 and 2010, 24,239 samples were analyzed, and 9,973 (41.1%) possessed at least one respiratory virus. EVs were found in 175 samples (0.7%). Our results revealed a clear predominance of EVB species, 90.9% (159/175). No EVDs were isolated. The mean and median ages of EV-positive subjects were 9.1 and 4.0 years, respectively, much younger than the population sampled, 17.6 and 12.0 years. Sixteen serotypes were identified, four EVA, 11 EVB, and one EVC species. The most common serotypes were coxsackievirus B1, coxsackievirus B2, coxsackievirus B5, and coxsackievirus B3.

**Conclusion:**

This study provides data about the serotypes of EVs circulating in Peru and sets the need for further studies.

## Background

Acute respiratory infections (ARIs) are a significant source of morbidity and mortality worldwide and disproportionately affect children, who have an average of two to seven ARIs each year [1]. Enteroviruses (EVs) ―family *Picornaviridae,* genus *Enterovirus*―are small, non-enveloped, and possess a single-stranded positive (messenger)-sense RNA genome of ~7.4 kb. Historically, these viruses were characterized by physical features such as stability or lability to acid pH, insensitivity to nonionic detergents, and resistance to ether, chloroform, and alcohol. For the most part, molecular characterization and taxonomic analysis of picornavirus genomes have replaced physical characterization and has led to the current classification scheme [2–4]. EVs are classified into seven species (EVA-D and rhinoviruses [RHVs] A-C), according to their genotypic and antigenic characteristics. Throughout the remainder of this report, EV will refer to only EVA, EVB, EVC, and EVD species and not the RHV species.

EVs are transmitted mainly from person to person by fecal-oral or oral-oral routes and by contact with upper respiratory secretions, fomites, or fluid from blisters [2, 5]. Although most EV infections remain asymptomatic, they are also responsible for a wide range of clinical syndromes, such as aseptic meningitis, encephalitis, myocarditis, acute flaccid paralysis, hand-foot-and-mouth disease, and herpangina [2, 3, 6, 7].

EVs have been isolated during influenza-like illnesses (ILI) surveillance studies that our team and others have conducted [8–13]. During the 2009 pandemic of influenza A virus (pH1N1), coxsackievirus (CV) and echovirus (E) were the most common viral pathogens in pH1N1-negative samples [14]. Although EV serotypes can co-circulate, different predominant serotypes are observed in different regions: for example, in France [15] and Spain [16], E11 and E6; in Taiwan [17] and China [18], CVB3 and CVA21; and in Brazil [19], E11. Epidemiological surveillance provides important information to understand the changing patterns of EV circulation and disease association. Accurate identification of the EV serotype may provide relevant epidemiological information such as the cause of a localized outbreak or the dominant EV circulating each year or it may be used to detect new serotypes or variants. Our current understanding of the role of EVs in respiratory infections in South America is restricted to prevalence data in some surveillance studies of ILI or ARI [8–11, 19], with only one report of specific EV serotypes associated with ARIs [19]. The purpose of this study was to detect, classify, and analyze the epidemiologic characteristics of EVs isolated from participants with ILI in Peru between 2005 and 2010.

## Results

### General findings

During the 6-year study period, throat swabs were collected from 24,239 participants aged 0-100 years (median 12.0 years, mean 17.6 years); 53.7% of the participants were less than 15 years. Among the 11 provinces included in this study, four accounted for more than 71.0% of the samples. These were Piura (23.4%), Loreto (21.0%), Lima (14.1%), and Tumbes (13.4%). Moreover, 33.0% of the specimens were collected in 2009 (year of pH1N1; Table 1).

**Table 1:** **Demographics and virus breakdown of subjects with influenza-like illness and enterovirus (EV) recovered from a pharyngeal sample**

			EVA				EVB											EVC
	Samples collected (n = 24,239)	EVs (n = 175)	CVA4 (n = 1)	CVA6 (n = 1)	CVA16 (n = 7)	EV71 (n = 4)	CVB1 (n = 84)	CVB2 (n = 31)	CVB3 (n = 12)	CVB4 (n = 2)	CVB5 (n = 15)	CVA9 (n = 1)	E3 (n = 1)	E4 (n = 3)	E9 (n = 8)	E13 (n = 1)	E30 (n = 1)	PV1 (n = 3)
Age group																		
0-4 years	7,382	90	1	1	6	4	32	17	5	2	7	1	-	3	7	1	-	3
5-9 years	3,293	32	-	-	1	-	13	10	2	-	4	-	-	-	1	-	1	-
10-14 years	2,355	22	-	-	-	-	15	4	1	-	2	-	-	-	-	-	-	-
15-19 years	2,440	11	-	-	-	-	7	-	1	-	2	-	1	-	-	-	-	-
20-29 years	3,557	7	-	-	-	-	6	-	1	-	-	-	-	-	-	-	-	-
30-39 years	2,090	7	-	-	-	-	5	-	2	-	-	-	-	-	-	-	-	-
40-49 years	1,364	2	-	-	-	-	2	-	-	-	-	-	-	-	-	-	-	-
≥ 50 years	1,688	4	-	-	-	-	4	-	-	-	-	-	-	-	-	-	-	-
missing	70	-	-	-	-	-	-	-	-	-	-	-	-	-	-	-	-	-
Collection province																		
***Coast***																		
Tumbes	3,256	23	-	-	-	-	7	9	-	1	1	-	-	-	5	-	-	-
Piura	5,661	67	-	1	3	2	25	11	7	1	5	1	1	3	3	1	-	3
La Libertad	1,034	8	1	-	-	-	4	-	1	-	2	-	-	-	-	-	1	-
Lima	3,418	23	-	-	2	-	14	3	3	-	-	-	-	-	-	-	-	-
***Southern highlands***																		
Arequipa	992	3	-	-	1	-	1	1	-	-	-	-	-	-	-	-	-	-
Cusco	1,412	3	-	-	-	-	3	-	-	-	-	-	-	-	-	-	-	-
Puno	679	3	-	-	-	-	3	-	-	-	-	-	-	-	-	-	-	-
***Jungle***																		
Loreto	5,082	32	-	-	1	2	20	3	1	-	5	-	-	-	-	-	-	-
Ucayali	1,131	9	-	-	-	-	4	4	-	-	1	-	-	-	-	-	-	-
Madre de Dios	604	1	-	-	-	-	1	-	-	-	-	-	-	-	-	-	-	-
Junin	970	3	-	-	-	-	2	-	-	-	1	-	-	-	-	-	-	-
Loreto	5,082	32	-	-	1	2	20	3	1	-	5	-	-	-	-	-	-	-
Collection year																		
2005	955	19	-	1	-	-	11	5	-	-	3	-	-	-	-	-	-	-
2006	2,076	28	-	-	1	1	14	7	1	-	-	-	-	1	2	-	-	-
2007	3,503	8	-	-	-	-	3	-	1	-	-	1	-	-	3	-	-	-
2008	3,699	23	1	-	2	1	2	6	4	-	6	-	-	-	1	-	-	1
2009	7,998	77	-	-	4	2	42	12	3	-	5	-	1	2	2	1	1	1
2010	6,008	20	-	-	-	-	12	1	3	2	1	-	-	-	-	-	-	1

EVA: enterovirus A; EVB: enterovirus B; EVC: enterovirus C; CV: coxsackievirus, E: echovirus; PV: poliovirus.

At least one respiratory virus was detected in 9,973 (41.1%) specimens, with influenza A virus being the most prevalent pathogen (62.3%). EVs were isolated in 175 participants (0.7% of samples taken) aged 0-89 years (median 4.0 years, mean 9.1 years). Rhesus monkey kidney cells (LLCMK2) identified six of the 13 EVA isolates, 152 of the 159 EVB isolates, and all three of the EVC isolates, while African green monkey kidney cells (Vero E6) identified 12 of the 13 EVAs, 115 of the 159 EVBs, and none of the EVCs.

Children under 15 years were the group with highest proportion of EVs detected (144/13,030; 1.1%) compared with the proportion found in the group of subjects ≥15 years (31/11,139; 0.3%), *X*^2^ = 53.3, p < 0.001. The male/female ratio for EV infection was 1.3, a ratio (1.1) similar to the study population (*X*^2^ = 0.84, p > 0.05). Also, our findings revealed that provinces located on the coast had the highest proportion of EV cases (121/13,369; 0.9%), followed by the samples collected from sites located in the jungle (45/7,787; 0.6%) and the southern highlands (9/3,083; 0.3%), (*X*^2^ = 15.1, p < 0.001).

### Enterovirus species in Peru

Of the 175 EVs isolated, 13 (7.4%) were EVA species, 159 (90.9%) were EVB species, and the remaining three (1.7%) were EVC species. The three EVC isolates were all related to the poliovirus (PV) Sabin vaccine strain, PV1. EVD species were not isolated in our study. EVA and EVC species were restricted entirely to children between five months and six years of age (median 1.5 years, mean 1.9 years).On the other hand, EVB species were detected from participants 0-89 years of age (median 5.0 years, mean 9.7 years). Table 1 shows the distribution of EV serotypes by age group, collection province, and year. Sixteen different EV serotypes were found: four EVA, 11 EVB, and one EVC serotype. The four main serotypes isolated were CVB1 (48.0%), CVB2 (17.7%), CVB5 (8.6%), and CVB3 (6.9%). EVs such as EV71, CVA4, CVA6, E4, CVB4, CVA9, E30, and PV1 were isolated exclusively in children less than five years. Fifteen of the 17 serotypes identified in this study were found in Piura, four of which were isolated only there. Moreover, Loreto and Tumbes had six and five serotypes, respectively.

Other respiratory viruses were co-detected with EV in 42 (24.0%) samples, 40 samples with one virus (influenza A virus [n = 16], herpes simplex virus, [n = 11], adenovirus [n = 9], parainfluenza virus 1 [n = 2], influenza B virus [n = 1], or respiratory syncytial virus [n = 1]) and two samples with two viruses (influenza A virus/herpes simplex virus [n = 1] and influenza B virus/human metapneumovirus [n = 1]). At the time of study enrollment, the EV cases had a median duration of illness of two days. Besides the inclusion criteria symptoms ― fever (96.0%), cough (83.1%), and sore throat (68.8%) ― participants presented with other common symptoms such as malaise (81.5%), rhinorrhea (72.7%), and headache (57.8%). No difference was found between EVA and EVB cases with respect to duration of illness, median axillary temperature, and symptoms.

### Phylogenetic analyses of EV in Peru

**Figure 1:**
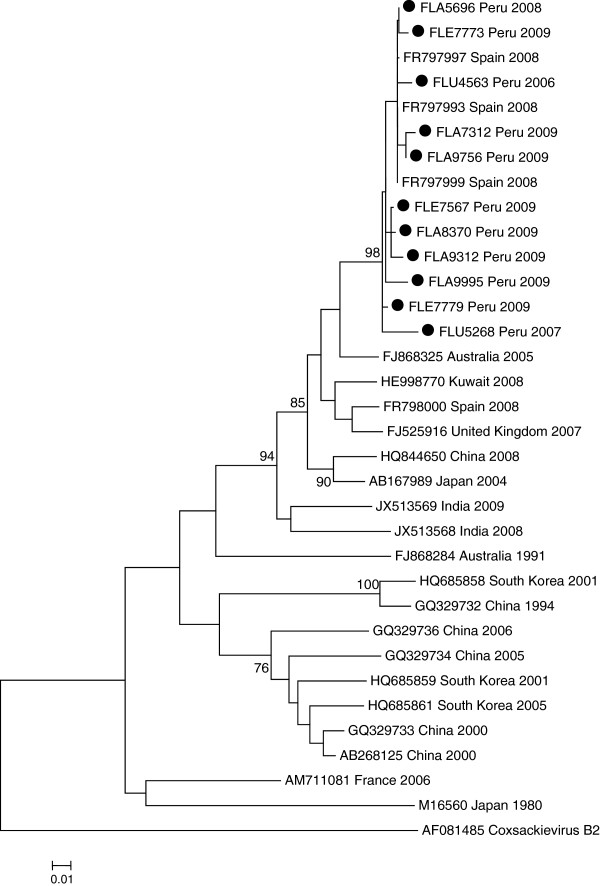
**Phylogenetic analysis of CVB1.** Phylogenetic analysis based on partial (234 bp) VP1 sequences. Only bootstrap values >75% are shown at nodes. Phylogenetic relationship between Peru coxsackievirus B1 (CVB1) (●) and other CVB1 sequences available from the GenBank database. All Peru strains clustered with Spain strains isolated in 2008. Sequences were named: accession number, country/province, and year. The prototypic CVB1, a strain from Japan, was included in the tree. CVB2 was used to root the tree.

Samples that were assigned the same serotype by BLAST analysis clustered with their homologous prototype strain, confirming serotype designation and supported by high bootstrap values. In addition, to investigate the genetic relationship among CVB1 strains (the predominant serotype in our study), we performed phylogenetic analysis, including sequences from the GenBank database of different geographical regions and year of isolation. Our CVB1 isolates clustered together (nucleotide identities 99.6 – 99.9%) and were phylogenetically close to isolates circulating in Spain during 2008 [7] and less related to Asian strains (Figure 1).

## Discussion

This study represents a retrospective analysis of the different EV serotypes detected in ILI cases in Peru over a 6-year period and complements the sparse existing data for the country [8–10, 13]. Our findings indicate that EVs were isolated more frequently in children younger than 5 years, similar to other studies that also examined EVs in older children or adults, including investigations from Spain, France, and Peru [7, 13, 15, 16]. Furthermore, the predominance of EVB in our study--representing 90% of all EV isolates--is in concordance with other studies that have shown EVB species as the main EV associated with ARIs [15, 17, 19].

Three PV1 isolates were also observed in this study all related to the Sabin vaccine strain. Although wild-type PV infection has been eliminated from South America and much of the world, vaccine-derived polioviruses still may have public health implications. These viruses may cause polio outbreaks in areas with low vaccine coverage, can replicate for years in persons who are immunodeficient, and may rarely cause paralysis in those with no known immunodeficiency[20].

Our regional results revealed that the coastal region, in particular Piura, had the highest EV isolation rate. Also, this province provided most of the isolations (Table 1), with the majority occurring from November through March. These findings may have been influenced by climatic conditions, which are notable for a rainy season during the summer (December – March). Others have noted a similar correlation with either the summer or rainy season [2, 4].

Our CVB1 isolates were closely related to one another, suggesting that a single strain was responsible for the cases of CVB1 that occurred throughout Peru from 2005 – 2010. Phylogenetically, our CVB1 strains possessed similarity to an isolate from Australia detected in 2005 (found in GenBank with no reference cited) and an even closer relation to isolates circulating in Spain that were isolated in a hand-foot-and-mouth disease study during 2008 [7]. Asian strains, in particular from South Korea and China, seemed to be less related to our isolates.

A limitation of our study was the use of cell culture for initial EV identification. No cell line is capable of supporting the growth of all EVs, and the use of different cells is recommended for EV isolation [2, 21, 22]; however, there is no consensus about which one(s) should be used. Some investigators use at least four cell lines [21], and we employed Madin-Darby canine kidney cells (MDCK), LLCMK2, and Vero cells for routine isolation of respiratory viruses in our laboratory. However, detection and identification of EVs only occurred in LLCMK2 and Vero cells, most likely due to their established better sensitivity for EVs compared with MDCK [2, 21]. Although direct comparisons between LLCMK2 and Vero cells for EV isolation are uncommon, a few studies indicate that Vero cells support the growth of EVA well and LLCMK2 cells support the growth of EVB and EVC well, similar to our study [21, 23, 24]. Utilization of other cell lines—such as Buffalo green monkey kidney cells and continuous human diploid fibroblasts—may have further increased our sensitivity in detecting EVs [25], in particular those that may not have grown well on the cell lines used in our study.

Initial use of PCR directly on the specimens would have probably increased the detection of EVs, as shown in an aforementioned study in Peru that detected EVs in 3% of respiratory samples [13]. It would have also allowed a better comparison between the different cell culture types. When used in the healthcare setting, PCR may better elucidate the role and frequency of EVs in central nervous system infections and hand-foot-and-mouth disease.

Another limitation was the passive surveillance nature of the study. Although this allowed us to collect a large number of samples, we were not able to get mild (i.e., not sick enough to go to a medical clinic) or severe (i.e., admitted directly to the hospital) cases. Also, we evaluated our subjects at just one point in time which made it impossible to assess the duration or severity of the disease after a subject left the clinic.

**Figure 2:**
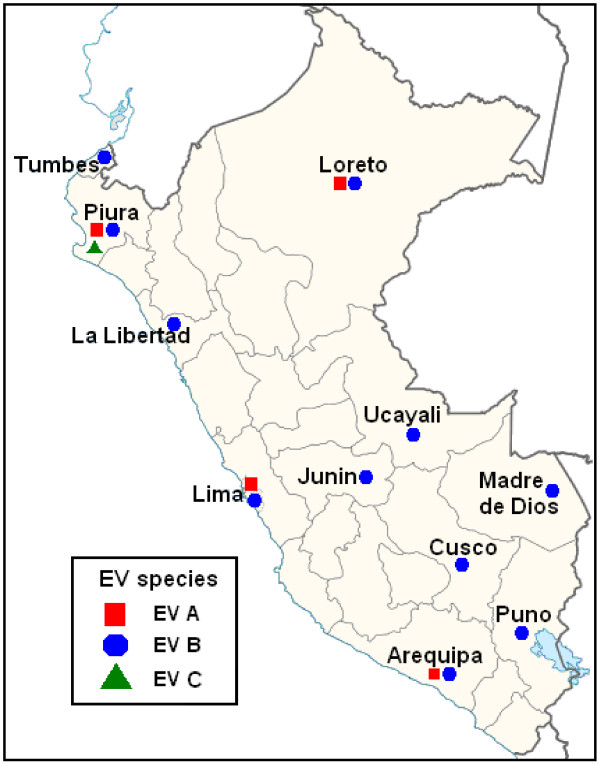
**Distribution of enterovirus species by location.** Peru (2005-2010).

In summary, our data reveal that EVs are commonly recovered from Peruvian children with ILI. Moreover, our findings show the concomitant circulation of distinct EV species in four provinces (Figure 2). We hope that these results will stimulate further research of EV and ILI. Such studies will provide justification for development of diagnostic and treatment options for a virus that may account for a large fraction of ARIs, both within Peru and throughout the world.

## Methods

### Ethics

The study protocol (NMRCD.2002.0019) was classified as less than minimal risk and was approved by the institutional review board (IRB) of the U.S. Naval Medical Research Center in Silver Spring, Maryland. Local government approval was obtained to conduct the study. This study was part of a respiratory virus surveillance network conducted by the Peruvian MoH in collaboration with NAMRU-6. Verbal consent was obtained from all participants using an information sheet approved and stamped by the NMRC IRB.

### Specimen and data collection

ILI was defined as axillary temperature (≥37.5°C) and cough and/or sore throat. Subjects of any age were allowed to participate. Samples collected from January 2005 through December 2010 were included in this study. Throat swab specimens were obtained by trained personnel using flocked swabs and placed immediately into a 3 ml viral transport media tube (UTM Diagnostic Hybrids; USA). Each specimen was stored at -70°C on site until delivery on dry ice to NAMRU-6 for laboratory analysis. Samples analyzed in this study were collected in health facilities located in 11 provinces divided in three regions: coast, southern highlands, and jungle (Figure 2, Table 1).

### Cell culture isolation and virus identification

All samples were inoculated onto three cell lines obtained from the American Type Culture Collection (ATCC®): Madin-Darby canine kidney cells (MDCK; CCL-34^TM^), African green monkey kidney cells (Vero E6; CRL-1586^TM^), and Rhesus monkey kidney cells (LLCMK2; CCL-7^TM^). Either after 10 days of inoculation or once cytopathic effect was observed, the presence of respiratory viral antigens was tested in all samples by immunofluorescence using commercial monoclonal antibodies (Diagnostic Hybrids; USA). Our immunofluorescence assay (IFA) used a blend of monoclonal antibodies for EV (Diagnostics Hybrids; USA) and specific monoclonal antibodies for 18 serotypes (Millipore; USA); PV1-3; CVA9, CVA16, and CVA24; CVB1-6; E4, E6, E9, E11, E30, and EV71. All assays were performed following the manufacturers’ established protocols. Besides EVs, viral antigens for adenovirus, influenza A virus, influenza B virus, parainfluenza viruses 1-3, respiratory syncytial virus, herpes simplex virus, and human metapneumovirus were tested by IFA (Diagnostics Hybrids; USA).

### RNA extraction, PCR, and sequencing

EVs detected with the blend of monoclonal antibodies and with negative results for specific group and/or serotype testing were selected for classification by molecular methods. RNA was extracted from cell culture supernatant using the Viral RNA Mini Kit (QIAamp, Qiagen; USA), according to the manufacturer’s recommendations. For EV typing, a semi-nested PCR that amplified part of the viral protein 1 (VP1) gene was carried out using a previously described method [26]. For direct sequencing, gene fragments were amplified and sequenced using the Big Dye terminator cycle sequencing kit version 3.1 (Applied Biosystems; USA) on an 3130XL DNA Sequencer (Applied Biosystems; USA). Nucleotide sequences of PCR products were analyzed by sequencing using Sequencher 4.8 software (Applied Biosystems; USA) and BioEdit software version 7.0.0 (Isis Pharmaceuticals Inc.; USA). PVs isolated in this study were further analyzed at the Centers for Disease Control and Prevention (Atlanta, Georgia) by intratypic differentiation real-time PCR and vaccine derived poliovirus real-time PCR.

### Phylogenetic analyses

To determinate the serotype, the VP1 sequences obtained were compared pairwise with sequences reported in the GenBank database through the BLAST search system. According to the results of this comparison, the EV detected in a sample was assigned to a serotype if it shared ≥75% nucleotide or ≥88% amino acid sequence identity [27]. Moreover, to illustrate the relationship between sequences from our most common EV serotype, CVB1, and CVB1 isolates from other locations in the world, we performed phylogenetic analysis on a partial 234 nucleotide segment of the VP1 gene. Multiple sequence alignments were performed by the Clustal program in the Mac Vector software package (Mac Vector Inc.; USA). Genetic distances were calculated using the Kimura 2-parameter model of nucleotide substitution and the reliability of the phylogenies was estimated by bootstrap analysis with 1,000 replicates. Phylogenetic trees were reconstructed by the neighbor-joining algorithm, using the MEGA 5.05 software.

### Accession numbers

Eleven partial VP1 genome sequences of CVB1 obtained in this study have been deposited in the GenBank database under accession numbers: KF962544 – KF962554.

### Statistical analyses

All the data from forms and laboratory results were entered using Microsoft Office Access 2003. Proportions were compared using Pearson Chi-Square test. A two-tailed critical value of alpha = 0.05 was used for statistical analysis using the SPSS Statistics software version 17.0 (SPSS Inc; USA). Although there was an overlap of four months between this and a prior study from our group [13], no sample was used in both studies.

## References

[1] Monto AS (2002). Epidemiology of viral respiratory infections. Am J Med.

[2] Romero JR, Murray PR, Baron EJ, Jorgensen MA, Pfaller MA, Landry ML (2007). Enteroviruses and Parechoviruses. Manual of Clinical Microbiology.

[3] Minor PD, Muir P, Zuckerman AJ, Banatvala JE, Schoub BD, Griffiths PD, Mortimer P (2009). Enteroviruses. Principles and Practice of Clinical Virology.

[4] Modlin J, Mandell G, Bennet J, Dolin R (2010). Introduction to the Enteroviruses and Parechoviruses. Mandell, Douglas, and Bennett’s Principles and Practice of Infectious Diseases.

[5] Wikswo ME, Khetsuriani N, Fowlkes AL, Zheng X, Penaranda S, Verma N, Shulman ST, Sircar K, Robinson CC, Schmidt T, Schnurr D, Oberste MS (2009). Increased activity of Coxsackievirus B1 strains associated with severe disease among young infants in the United States, 2007-2008. Clin Infect Dis.

[6] Park K, Lee B, Baek K, Cheon D, Yeo S, Park J, Soh J, Cheon H, Yoon K, Choi Y (2012). Enteroviruses isolated from herpangina and hand-foot-and-mouth disease in Korean children. Virol J.

[7] Bracho MA, Gonzalez-Candelas F, Valero A, Cordoba J, Salazar A (2011). Enterovirus co-infections and onychomadesis after hand, foot, and mouth disease, Spain, 2008. Emerg Infect Dis.

[8] Laguna-Torres VA, Gomez J, Ocana V, Aguilar P, Saldarriaga T, Chavez E, Perez J, Zamalloa H, Forshey B, Paz I, Gomez E, Ore R, Chauca G, Ortiz E, Villaran M, Vilcarromero S, Rocha C, Chincha O, Jimenez G, Villanueva M, Pozo E, Aspajo J, Kochel T (2009). Influenza-like illness sentinel surveillance in Peru. PLoS One.

[9] Laguna-Torres VA, Sanchez-Largaespada JF, Lorenzana I, Forshey B, Aguilar P, Jimenez M, Parrales E, Rodriguez F, Garcia J, Jimenez I, Rivera M, Perez J, Sovero M, Rios J, Gamero ME, Halsey ES, Kochel TJ (2011). Influenza and other respiratory viruses in three Central American countries. Influenza Other Respir Viruses.

[10] Laguna-Torres VA, Gomez J, Aguilar PV, Ampuero JS, Munayco C, Ocana V, Perez J, Gamero ME, Arrasco JC, Paz I, Chavez E, Cruz R, Chavez J, Mendocilla S, Gomez E, Antigoni J, Gonzalez S, Tejada C, Chowell G, Kochel TJ (2010). Changes in the viral distribution pattern after the appearance of the novel influenza A H1N1 (pH1N1) virus in influenza-like illness patients in Peru. PLoS One.

[11] Douce RW, Aleman W, Chicaiza-Ayala W, Madrid C, Sovero M, Delgado F, Rodas M, Ampuero J, Chauca G, Perez J, Garcia J, Kochel T, Halsey ES, Laguna-Torres VA (2011). Sentinel surveillance of influenza-like-illness in two cities of the tropical country of Ecuador: 2006-2010. PLoS One.

[12] Tokarz R, Kapoor V, Wu W, Lurio J, Jain K, Mostashari F, Briese T, Lipkin WI (2011). Longitudinal molecular microbial analysis of influenza-like illness in New York City, May 2009 through May 2010. Virol J.

[13] Garcia J, Espejo V, Nelson M, Sovero M, Villaran MV, Gomez J, Barrantes M, Sanchez F, Comach G, Arango AE, Aguayo N, de Rivera IL, Chicaiza W, Jimenez M, Aleman W, Rodriguez F, Gonzales MS, Kochel TJ, Halsey ES (2013). Human rhinoviruses and enteroviruses in influenza-like illness in Latin America. Virol J.

[14] Koon K, Sanders CM, Green J, Malone L, White H, Zayas D, Miller R, Lu S, Han J (2010). Co-detection of pandemic (H1N1) 2009 virus and other respiratory pathogens. Emerg Infect Dis.

[15] Jacques J, Moret H, Minette D, Leveque N, Jovenin N, Deslee G, Lebargy F, Motte J, Andreoletti L (2008). Epidemiological, molecular, and clinical features of enterovirus respiratory infections in French children between 1999 and 2005. J Clin Microbiol.

[16] Trallero G, Avellon A, Otero A, De Miguel T, Perez C, Rabella N, Rubio G, Echevarria JE, Cabrerizo M (2010). Enteroviruses in Spain over the decade 1998-2007: virological and epidemiological studies. J Clin Virol.

[17] Lo CW, Wu KG, Lin MC, Chen CJ, Ho DM, Tang RB, Chan YJ (2010). Application of a molecular method for the classification of human enteroviruses and its correlation with clinical manifestations. J Microbiol Immunol Infect.

[18] Xiang Z, Gonzalez R, Wang Z, Ren L, Xiao Y, Li J, Li Y, Vernet G, Paranhos-Baccala G, Jin Q, Wang J (2012). Coxsackievirus A21, enterovirus 68, and acute respiratory tract infection, China. Emerg Infect Dis.

[19] Portes SA, Da Silva EE, Siqueira MM, De Filippis AM, Krawczuk MM, Nascimento JP (1998). Enteroviruses isolated from patients with acute respiratory infections during seven years in Rio de Janeiro (1985-1991). Rev Inst Med Trop Sao Paulo.

[20] Centers for Disease Control and Prevention (CDC) (2012). Update on vaccine-derived polioviruses--worldwide, April 2011-June 2012. MMWR Morb Mortal Wkly Rep.

[21] She RC, Crist G, Billetdeaux E, Langer J, Petti CA (2006). Comparison of multiple shell vial cell lines for isolation of enteroviruses: a national perspective. J Clin Virol.

[22] Terletskaia-Ladwig E, Meier S, Hahn R, Leinmuller M, Schneider F, Enders M (2008). A convenient rapid culture assay for the detection of enteroviruses in clinical samples: comparison with conventional cell culture and RT-PCR. J Med Microbiol.

[23] Mizuta K, Abiko C, Goto H, Murata T, Murayama S (2003). Enterovirus isolation from children with acute respiratory infections and presumptive identification by a modified microplate method. Int J Infect Dis.

[24] Mizuta K, Abiko C, Aoki Y, Suto A, Hoshina H, Itagaki T, Katsushima N, Matsuzaki Y, Hongo S, Noda M, Kimura H, Ootani K (2008). Analysis of monthly isolation of respiratory viruses from children by cell culture using a microplate method: a two-year study from 2004 to 2005 in yamagata, Japan. Jpn J Infect Dis.

[25] Chonmaitree T, Ford C, Sanders C, Lucia HL (1988). Comparison of cell cultures for rapid isolation of enteroviruses. J Clin Microbiol.

[26] Iturriza-Gomara M, Megson B, Gray J (2006). Molecular detection and characterization of human enteroviruses directly from clinical samples using RT-PCR and DNA sequencing. J Med Virol.

[27] Oberste MS, Maher K, Kilpatrick DR, Flemister MR, Brown BA, Pallansch MA (1999). Typing of human enteroviruses by partial sequencing of VP1. J Clin Microbiol.

